# The therapeutic potential of working memory training for treating mental disorders

**DOI:** 10.3389/fnhum.2015.00481

**Published:** 2015-09-03

**Authors:** Sharaf Ansari

**Affiliations:** Samsung R&D InstituteBangalore, India

**Keywords:** working memory, fluid intelligence, depression, ADHD, schizophrenia

Working memory (WM) is a measure of the number of items that can be held in short-term memory at once (Baddeley, 1992). Working memory is hypothesized to be a core component underlying fluid intelligence (Engle et al., 1999), and is the base ability tapped by a battery of complex task such as visuospatial reasoning and reading comprehension (Baddeley, 2003). It was once believed to be static in an individual and to have a strong heritable component (Sternberg, 2008). Recent studies such as Jaeggi et al. (2008), however, have challenged this view.

There has been a recent furor over the possibility that working memory training, of the order of a few weeks, could lead to significant increases in fluid intelligence. Several studies (Jaeggi et al., 2008) have reported transfer effects, yet others (Redick et al., 2013; Thompson et al., 2013) have not shown similar increases, even with highly similar experimental methodology. The factors mediating transfer effects are still unclear. Recently, though it has been suggested (Jaeggi et al., 2014; Au et al., 2015) that individual differences such as intrinsic motivation and self-perceived cognitive deficits of the participants may play a role. The same factors could plausibly mediate the therapeutic benefits of working memory training in patients suffering from mental disorders.

The effects of training working memory are still being hotly debated. The evidence for transfer effects is still preliminary. There are several recent reviews of the literature such as Shipstead et al. (2012) and Melby-Lervåg and Hulme (2013) which offer detailed criticisms of reported transfer. Existing studies may suffer from a variety of flaws like an inadequate conceptualization of working memory, use of no-contact control groups, and failing to account for improvements in non-WM cognitive function. For a meta-analysis of the effects of working memory training which tries to address some of these concerns, see Au et al. (2015). For critical replies to this review, see Melby-Lervåg and Hulme (2015) and Bogg and Lasecki (2014). For the purposes of this paper, I take the position that some initial evidence of the effectiveness of working memory training exists.

There has been an interest in the value of working memory training for purposes other than directly inducing an increase in fluid intelligence, such as its potential to treat rumination in depression (Wanmaker et al., 2015), treating attention deficit hyperactivity disorder (Klingberg et al., 2002), and even improving deductive reasoning (Beatty and Vartanian, 2015). Also, there has been a growing interest and excitement about cognitive training for treating psychiatric disorders, for example Subramaniam and Vinogradov (2013).

With regards to the brain, working memory has been hypothesized to have a substrate in regions of the brain like the pre-frontal cortex, the most evolutionarily recent part of our brain. (D'Esposito et al., 1995) Its dysfunction is also suspected to be involved in the occurrence of several mental disorders such as depression, attention deficit hyperactivity disorder (ADHD) and schizophrenia.

Though not much research has been done on the brain changes caused by attempts to train working memory (and the research done varies wildly in methodology), preliminary research seems very encouraging. A review by Buschkuehl et al. (2012) found that structural and functional changes take place in the brain after working memory training and that improvements in working memory may be due to changes in activation in the prefrontal and parietal brain areas. So, working memory training may induce changes in brain regions believed to be dysfunctional in certain mental disorders.

Attention deficit hyperactivity disorder is a disorder characterized by impulsivity, inattention and inappropriate motor control (Klingberg et al., 2002; Polanczyk et al., 2007). It is one of the most common causes of learning disability in the world, affecting between 1 and 20% of all school children (Polanczyk et al., 2007).

Working memory is believed to be compromised in ADHD, possibly due to impairment in the frontal lobe (Schweitzer et al., 2000). Klingberg et al. (2002) found that working memory significantly increased performance on both trained and untrained WM tasks, in children suffering from ADHD. More recently, Beck et al. (2010) found that 5 weeks of working memory training in children and adolescents decreased measures of inattention, decreased number of ADHD symptoms, and improved working memory. This strongly suggests potential for working memory training to alleviate symptoms of ADHD. However the evidence for treating ADHD with working memory training is still severely lacking. Recent meta-analysis such as Cortese et al. (2015) and Sonuga-Barke et al. (2013) have not found reduction in ADHD symptoms, which implies that existing working memory training techniques may be of limited use in treating ADHD. A preliminary study on adolescents by Stevens et al. (2015) reported a normalization of deficits in frontoparietal brain region activation in ADHD patients and explores the possibility that cognitive training specifically tailored for treating ADHD might be more effective than the commonly used CogMed™ approach.

As an extremely common mood disorder, depression is a global phenomenon. It can be crippling in nature and has very high present and projected costs for society (Sobocki et al., 2006). Depression is also associated with impairments in working memory (Rose and Ebmeier, 2006), possibly due to constant inference to normal functioning from negative material (Joormann and Gotlib, 2008).

In a study to observe the effects of working memory training on rumination (a common symptom of depression), Onraedt and Koster (2014) found that 6 days of working memory training did not have a measurable effect on measures of depression. In another recent study Wanmaker et al. (2015) did not find any positive effect of working memory training on measures of depression, anxiety and rumination.

Even though these two studies did not show any alleviation of disease symptoms, it may be premature to assume the inefficacy of working memory training to treat depressed individuals. If intrinsic motivation plays a mediating role in guiding the efficacy of working memory training, it is plausible to suggest that the lack of positive effect in depressed patients could be due to a lack of motivation, a lack of interest being a key part of depression. Working memory training, combined with other non-pharmacological therapies such as an exercise regime, may still hold promise.

Schizophrenia is often associated with cognitive impairments, with a majority of patients having some sort of impairment (O'Carroll, 2000). There is evidence for disturbed frontotemporal disruptions during working memory tasks in patients suffering from schizophrenia (Meyer-Lindenberg et al., 2001).

A study by Hubacher et al. (2013) found that 4 weeks of working memory training improved visual and verbal memory in patients with chronic schizophrenia. Another study by Subramaniam et al. (2014) found increased working memory 6 months after a 16 week working memory training regime. A recent meta-analysis by Li et al. (2015) found WM training activation changes in the brain of schizophrenics. These results are exciting due to the extensive cognitive impairments generally faced by schizophrenia patients and the resulting occupational and social costs.

In summary, it appears that working memory training shows promise (possibly used in a supplementary fashion to more traditional treatment options). It is possible that the effects of working memory training in treating mental disorders are mediated by factors such as intrinsic motivation, which may in turn limit effectiveness in disorders characterized by a lack of motivation, such as depression. In conclusion, much research remains to be done, but, at present, working memory training seems to have great therapeutic potential.

## Conflict of interest statement

The author declares that the research was conducted in the absence of any commercial or financial relationships that could be construed as a potential conflict of interest.

## References

[B1] Au J. Sheehan E. Tsai N. Duncan G. J. Buschkuehl M. Jaeggi S. M. (2015). Improving fluid intelligence with training on working memory: a meta-analysis. Psychon. Bull. Rev. 22, 366–377. 10.3758/s13423-014-0699-x 25102926

[B2] Baddeley A. (1992). Working memory. Science 255, 556–559. 10.1126/science.1736359 1736359

[B3] Baddeley A. (2003). Working memory: looking back and looking forward. Nat. Rev. Neurosci. 4, 829–839. 10.1038/nrn1201 14523382

[B4] Beatty E. L. Vartanian O. (2015). The prospects of working memory training for improving deductive reasoning. Front. Hum. Neurosci. 9:56. 10.3389/fnhum.2015.00056 25705189 PMC4319378

[B5] Beck S. J. Hanson C. A. Puffenberger S. S. Benninger K. L. Benninger W. B. (2010). A controlled trial of working memory training for children and adolescents with ADHD. J. Clin. Child Adolesc. Psychol. 39, 825–836. 10.1080/15374416.2010.517162 21058129

[B6] Bogg T. Lasecki L. (2014). Reliable gains? Evidence for substantially underpowered designs in studies of working memory training transfer to fluid intelligence. Front. Psychol. 5:1589. 10.3389/fpsyg.2014.01589 25657629 PMC4302828

[B7] Buschkuehl M. Jaeggi S. M. Jonides J. (2012). Neuronal effects following working memory training. Dev. Cogn. Neurosci. 2, S167–S179. 10.1016/j.dcn.2011.10.001 22682905 PMC6987667

[B8] Cortese S. Ferrin M. Brandeis D. Buitelaar J. Daley D. Dittmann R. W.. (2015). Cognitive training for attention-deficit/hyperactivity disorder: meta-analysis of clinical and neuropsychological outcomes from randomized controlled trials. J. Am. Acad. Child Adolesc. Psychiatry 54, 164–174. 10.1016/j.jaac.2014.12.010 25721181 PMC4382075

[B9] D'Esposito M. Detre J. A. Alsop D. C. Shin R. K. Atlas S. Grossman M. (1995). The neural basis of the central executive system of working memory. Nature 378, 279–281. Available online at: http://www.nature.com/nature/journal/v378/n6554/abs/378279a0.html 10.1038/378279a0 7477346

[B10] Engle R. W. Tuholski S. W. Laughlin J. E. Conway A. R. (1999). Working memory, short-term memory, and general fluid intelligence: a latent-variable approach. J. Exp. Psychol. Gen. 128:309. 10.1037/0096-3445.128.3.309 10513398

[B11] Hubacher M. Weiland M. Calabrese P. Stoppe G. Stöcklin M. Fischer-Barnicol D. (2013). Working memory training in patients with chronic schizophrenia: a pilot study. 2013:154867. 10.1155/2013/154867 24236272 PMC3820077

[B12] Jaeggi S. M. Buschkuehl M. Jonides J. Perrig W. J. (2008). Improving fluid intelligence with training on working memory. Proc. Natl. Acad. Sci. U.S.A. 105, 6829–6833. 10.1073/pnas.0801268105 18443283 PMC2383929

[B13] Jaeggi S. M. Buschkuehl M. Shah P. Jonides J. (2014). The role of individual differences in cognitive training and transfer. Mem. Cognit. 42, 464–480. 10.3758/s13421-013-0364-z 24081919

[B14] Joormann J. Gotlib I. H. (2008). Updating the contents of working memory in depression: interference from irrelevant negative material. J. Abnorm. Psychol. 117, 182. 10.1037/0021-843X.117.1.182 18266496

[B15] Klingberg T. Forssberg H. Westerberg H. (2002). Training of working memory in children with ADHD. J. Clin. Exp. Neuropsychol. 24, 781–791. 10.1076/jcen.24.6.781.8395 12424652

[B16] Li X. Xiao Y. H. Zhao Q. Leung A. W. Cheung E. F. Chan R. C. (2015). The neuroplastic effect of working memory training in healthy volunteers and patients with schizophrenia: implications for cognitive rehabilitation. Neuropsychologia 75, 149–162. 10.1016/j.neuropsychologia.2015.05.029 26032579

[B17] Melby-Lervåg M. Hulme C. (2013). Is working memory training effective? A meta-analytic review. Dev. Psychol. 49, 270. 10.1037/a0028228 22612437

[B18] Melby-Lervåg M. Hulme C. (2015). There is no convincing evidence that working memory training is effective: a reply to Au et al. (2014) and Karbach and Verhaeghen (2014). Psychon. Bull. Rev. [Epub ahead of print]. Available online at: http://rd.springer.com/article/10.3758/s13423-015-0862-z 10.3758/s13423-015-0862-z 26082279

[B19] Meyer-Lindenberg A. Poline J. B. Kohn P. D. Holt J. L. Egan M. F. Weinberger D. R.. (2001). Evidence for abnormal cortical functional connectivity during working memory in schizophrenia. Am. J. Psychiatry 158, 1809–1817. 10.1176/appi.ajp.158.11.1809 11691686

[B20] O'Carroll R. (2000). Cognitive impairment in schizophrenia. Adv. Psychiatric Treat. 6, 161–168. 10.1192/apt.6.3.161

[B21] Onraedt T. Koster E. H. (2014). Training working memory to reduce rumination. PLoS ONE 9:e90632. 10.1371/journal.pone.0090632 24595102 PMC3940909

[B22] Polanczyk G. de Lima M. S. Horta B. L. Biederman J. Rohde L. A. (2007). The worldwide prevalence of ADHD: a systematic review and metaregression analysis. Am. J. Psychiatry 164, 942–948. 10.1176/appi.ajp.164.6.942 17541055

[B23] Redick T. S. Shipstead Z. Harrison T. L. Hicks K. L. Fried D. E. Hambrick D. Z.. (2013). No evidence of intelligence improvement after working memory training: a randomized, placebo-controlled study. J. Exp. Psychol. 142, 359. 10.1037/a0029082 22708717

[B24] Rose E. J. Ebmeier K. P. (2006). Pattern of impaired working memory during major depression. J. Affect. Disord. 90, 149–161. 10.1016/j.jad.2005.11.003 16364451

[B25] Schweitzer J. B. Faber T. L. Grafton S. T. Tune L. E. Hoffman J. M. Kilts C. D. (2000). Alterations in the functional anatomy of working memory in adult attention deficit hyperactivity disorder. Am. J. Psychiatry 157, 278–280. 10.1176/appi.ajp.157.2.278 10671402

[B26] Shipstead Z. Redick T. S. Engle R. W. (2012). Is working memory training effective? Psychol. Bull. 138, 628. 10.1037/a0027473 22409508

[B27] Sobocki P. Jönsson B. Angst J. Rehnberg C. (2006). Cost of depression in Europe. J. Ment. Health Policy Econ. 9, 87–98. Available online at: http://europepmc.org/abstract/med/17007486 17007486

[B28] Sonuga-Barke E. J. Brandeis D. Cortese S. Daley D. Ferrin M. Holtmann M.. (2013). Nonpharmacological interventions for ADHD: systematic review and meta-analyses of randomized controlled trials of dietary and psychological treatments. Am. J. Psychiatry. 170, 275–289. Available online at: http://ajp.psychiatryonline.org/doi/10.1176/appi.ajp.2012.12070991 10.1176/appi.ajp.2012.12070991 23360949

[B29] Sternberg R. J. (2008). Increasing fluid intelligence is possible after all. Proc. Natl. Acad. Sci. U.S.A. 105, 6791–6792. 10.1073/pnas.0803396105 18474863 PMC2383939

[B30] Stevens M. C. Gaynor A. Bessette K. L. Pearlson G. D. (2015). A preliminary study of the effects of working memory training on brain function. Brain Imaging behav. [Epub ahead of print]. Available online at: http://rd.springer.com/article/10.1007/s11682-015-9416-2page-1 10.1007/s11682-015-9416-2 PMC4698365 26138580

[B31] Subramaniam K. Luks T. L. Garrett C. Chung C. Fisher M. Nagarajan S.. (2014). Intensive cognitive training in schizophrenia enhances working memory and associated prefrontal cortical efficiency in a manner that drives long-term functional gains. Neuroimage 99, 281–292. 10.1016/j.neuroimage.2014.05.057 24867353 PMC4498800

[B32] Subramaniam K. Vinogradov S. (2013). Cognitive training for psychiatric disorders. Neuropsychopharmacology 38, 242–243. 10.1038/npp.2012.177 23147484 PMC3521981

[B33] Thompson T. W. Waskom M. L. Garel K. L. A. Cardenas-Iniguez C. Reynolds G. O. Winter R.. (2013). Failure of working memory training to enhance cognition or intelligence. PLoS ONE 8:e63614. 10.1371/journal.pone.0063614 23717453 PMC3661602

[B34] Wanmaker S. Geraerts E. Franken I. H. (2015). A working memory training to decrease rumination in depressed and anxious individuals: a double-blind randomized controlled trial. J. Affect. Disord. 175, 310–319. Available online at: http://www.sciencedirect.com/science/article/pii/S0165032714008131. 10.1016/j.jad.2014.12.027 25661397

